# Invisible Invertebrates: The Welfare of Invertebrates in Public Aquaria

**DOI:** 10.3390/ani13233620

**Published:** 2023-11-23

**Authors:** Kerry Perkins

**Affiliations:** Aquaculture, Fisheries and Marine Studies, University Centre Sparsholt, Winchester SO21 2NF, UK; kerry.perkins@sparsholt.ac.uk

**Keywords:** invertebrate, aquarium, welfare

## Abstract

**Simple Summary:**

Aquatic invertebrates are understudied and often overlooked in public aquarium welfare. By determining public opinion on aquatic invertebrate welfare, we can obtain a better picture on whether they are even considered during an aquarium visit. Using TripAdvisor, it was determined that aquatic invertebrates were under-represented in negative welfare comments compared with their vertebrate counterparts. The public were more likely to comment on welfare issues if from the UK or USA. To aid in a better understanding of aquatic welfare by the public, zoo and aquarium associations should help create guidelines for aquatic invertebrates. Guideline creation would help increase the construction of welfare indicators and support welfare tool kits. This would hopefully result in the better consideration of invertebrate welfare and public understanding in public aquaria.

**Abstract:**

Awareness of welfare issues within animal collections is increasing as information becomes more accessible for staff and the public. A knowledge gap remains when considering the welfare of invertebrates, particularly when housed in public aquaria. TripAdvisor comments were analyzed for 485 worldwide aquariums. The public focused on anthropogenic features or charismatic organisms within collections. Invertebrate welfare was only presented in 18% of negative welfare comments compared with the 51% of represented vertebrates and 31% of negative general welfare comments. The UK and USA reported a greater number of perceived invertebrate welfare issues. Greater dissemination of information between aquarists and scientists should be encouraged to drive welfare standards and improve husbandry. In addition, incorporating input from invertebrate aquarists while utilizing welfare toolkits are vital for improving overall standards if we are to have greater representation of invertebrate welfare in public aquaria.

## 1. Introduction

The definition of animal welfare has been considerably debated within the literature and is often adjusted depending on the lens of the observer [1,2,3]. Within zoos and aquariums, welfare is often based around Mellor’s five domains model [4] which has five domains split into nutrition, environment, health, behavior and mental state. Both the World Association of Zoos and Aquariums (WAZA) and the European Association of Zoos and Aquaria (EAZA) [5] have adopted Mellor’s model to monitor animal welfare status within collections. What is considered good animal welfare in a captive setting maybe different from wild animals due to different pressures acting on the animal [3]. Subsequently, priorities in an animal’s life may be altered if it is in a captive setting. Aspects such as life cycle, avoidance of predators and access to food may be considerably different compared to their wild counterparts. Therefore, the careful creation of environments within aquaria are important, as a large number of animals displayed are still captured from the wild [6].

Understanding of wild animal welfare and human input is still debated [1,2,3,7], particularly in species with limited knowledge of conditions in the wild and their life cycles. A captive environment may also contain different goals and husbandry settings than that of the wild. Within the UK, laboratory settings base animal numbers and welfare on the 3R model of replacement, reduction, and refinement, by either replacing the animal use with alternatives such as simulations, reducing the number of animals used or refining the experiment methodology. This reduces the overuse of animals within scientific research. Legislation also gives additional protection, welfare conditions and guidance on vertebrates within these situations [8,9]. Similarly, within zoo and aquarium populations, vertebrates are also given welfare considerations, often with a different focus, due to the conservation and breeding focus of many zoos [10]. In contrast, invertebrates do not necessarily receive the same consideration both in laboratory and zoo collections. Only some invertebrate groups such as cephalopods and decapods are protected in certain countries such as New Zealand [11,12]. This lack of consideration for invertebrates is often not due to lack of addressing the needs, but rather the lack of understanding that they have needs. 

With an estimated 97% of organisms being invertebrates, the overall understanding and research into their welfare despite their numbers is not comparable to that of vertebrates [13]. This is particularly evident when concerned with welfare indicators and positive/negative responses to stimuli. Debate on the pain perception of vertebrates and invertebrates has contributed to discussion on whether there is a capacity to suffer within certain species [14]. Recent studies in higher invertebrates such as cephalopods and crustaceans have shown the capacity for nociception [15,16]. Our understanding of pain experienced by invertebrates, however, is still limited [17]. This, therefore, raises the question around the potential capacity to suffer by invertebrates. The absence or difference of comparative organs such as a nervous system does not necessarily indicate a lack of negative or painful experiences for the organism [16]. Therefore, the ability to feel pain should not be the sole determining factor of poor welfare, particularly when using welfare models, and we should be cautious of using this metric alone.

Many animal welfare models are based off the requirements of terrestrial vertebrates. The five freedom model was developed from a 1965 UK government report on livestock husbandry with formalization in 1979 [18]. This key piece of guidance has given a base from which other models have been constructed. However, some aspects of the five freedoms such as ‘freedom from thirst’ have little relevance for aquatic species. Within the aquarium and zoo community, Mellor’s five domain model is the most popular [1]. Although useful for terrestrial vertebrates, its suitability for other species is still yet to be fully investigated [18,19]. Another criticism is that, like similar models, it is often based around a single point in time rather than the lifetime of experiences of an animal [20]. As a result, welfare tools based off the five domains have been constructed for utilization within zoos and aquaria instead. The Animal Welfare Assessment Grid (AWAG) has been tested in a variety of zoo and aquarium situations [20,21,22]. The AWAG, however, has not been trialed extensively within multispecies exhibits or with aquatic colonial species. 

Olge and DeSmet [7] suggest one way of improving welfare may be the inclusion of zookeepers in monitoring and assessment, and to use a more contemporary approach with a subjective measure of behavior and animal personality. There is, however, a question around whether zookeepers have sufficient understanding of welfare and whether it is a priority within their collection. It was suggested that by increasing staff utilization in the assessment, they may proactively meet the needs of the individual animal through the input of the animal staff. It is unclear, however, whether this would translate into an aquarium situation due to multiple species exhibits or potential bias arising towards vertebrates, such as prioritizing fish and mammals. It is, therefore, important to investigate the available resources to improve aquatic invertebrates and consider the current public perception of their welfare. 

A call to action by Oldfield and Bonano [22] highlighted the urgent need for zoos and aquaria to conduct behavior studies on bony fishes due to their under representation within the literature. Similarly, it is not through a lack of aquatic invertebrates within the collections, but the lack of study, which is reflected in the underrepresentation. Our custodial responsibilities to the animals are constantly improving and advancing; however, our improvements in welfare are still questionable [23]. In aquariums, this has particularly been evident with the increase in public interest documentaries such as Blackfish [24] and The Cove [25]. Historically, aquatic mammals were considered ambassadors for their species; however, due to recent documentaries, there is now a question around the validity of this claim [26]. With an increased awareness of and interest in welfare in aquariums, we should be encouraging better transparency of our welfare indicators. Better transparency may increase public confidence with collections, but also to allow the better understanding of behaviors that the organism may be exhibiting. One way to potentially standardize welfare monitoring is through tools provided by regional or global zoos and aquarium accreditation schemes.

Currently, the public awareness of zoo association programs is under-researched [27]. The understanding of aquarium welfare indicators by the public is also potentially limited, with most studies focusing on zoos [28]. Studies within zoo species have shown that the public often view certain behaviors as a lower standard of welfare, such as repetitive movement rather than resting [28,29]. Melfi et al. [30] also found that most visitors believe they understand animal welfare intuitively. When attending aquariums, the public’s motivation will vary and their preconceived ideas will be influenced by their existing knowledge and experience [22]. Prior experience combined with emotion can play an important role, with visitors experiencing a multi-layered zoo interaction as having a more positive perception [31]. Similar comments could be made about aquarists, who care for aquatic invertebrates, about how their existing history may influence overall day-to-day welfare decisions.

It is, therefore, important that we identify areas of potential influence and bias by the members of the public and potentially the aquariums themselves, if we are to fully understand welfare decisions. The rise of social media has given a platform for people to share their thoughts and views more freely [32]. Review sites have gained popularity as the world becomes more connected, giving people the opportunity to share their experiences [33]. By reviewing tourism businesses, the customers experience can be communicated to prospective patrons. Because of the ability to contribute to other people’s experience, websites such as Yelp and TripAdvisor have become a popular resource. Since its launch in 2000, TripAdvisor has acted as a guide to the public, but from 2004 user-generated content has been the main focus of the site, reaching 100’s of millions of visitors a month [34]. The popularity of the site has gained interest from researchers on sentiment analysis, complaints and satisfaction [32,33,34]. Because of the multiple aspects such as ethic, welfare and care comments, wildlife and tourism researchers have used TripAdvisor to understand the impact of wildlife encounters and drivers to ecotourism [35,36,37,38]. It is this investigation into welfare and public opinion of animal attractions that can aid us in understanding and potentially improving welfare within aquariums. In this article, we will investigate how the TripAdvisor platform may be used to gain insight into the public’s perception of welfare in aquarium. In addition, we will consider the potential actions collections can implement to assist in increasing invertebrate welfare knowledge of visitors.

## 2. Materials and Methods

A list of all aquariums on TripAdvisor was compiled in September 2020. This was conducted by utilizing the attractions function on TripAdvisor, which has a category for zoos and aquariums. In addition the search term, aquarium was inputted into the search function of the website to discover any additional aquariums that may have been missed due to not being included in the attractions category. Aquariums within Zoos and theme parks were excluded from analysis. This was due to the fact that keywords and comments around the aquarium could not be separated from other comments about the attraction. The location and number of reviews were recorded along with the rating of the aquarium in respect to the trip advisor scale of 1–5, (Terrible = 1, Poor = 2, Average = 3, Very good = 4, and Excellent = 5). Keywords of ‘Welfare’, ‘Dead’, ‘Sad’, ‘Horrible’, ‘Confinement’ and ‘Torture’ were then used to search the text for possible negative welfare implications. Keywords were selected from terms used by Walker et al. [39] as well as other terms that had been used by animal rights groups to explain aquarium welfare [40]. Only negative welfare comments were included due to the ambiguous and often unclear positive welfare comments within the text. Keywords were also translated into the recognized language of the aquarium’s country via google translate to reduce loss of data; this equated to 31 languages in total. The number of occurrences of the keyword were then recorded. Where the word was not used in respect to animal welfare, it was excluded from the data set. Results were broken down into major geographical regions determined by the location of the aquarium. In a large number of cases, the native language was the predominant number of reviews, suggesting that visitors were likely to be domestic to the country rather than international tourists.

Overall, comments were also analyzed around negative perceptions such as dirty tanks or tanks too small for the animal; the data set was then further refined to just include aquatic invertebrates for further analysis. The comments were divided by invertebrate species to identify differences. This also allowed for identifying other terms, such as sick, damaged and injured, that were not picked up in the key terms search specifically relating to invertebrates. ANOVAs were performed on the data in Minitab v20 between regions with Bonferroni post hoc tests to identify specific differences.

## 3. Results

### 3.1. Overall Visitor Satisfaction

A total of 485 aquariums were identified on TripAdvisor from 77 countries. The average rating for all aquariums was 3.95 out of 5, confirming a very good experience overall. However, there was variation between geographic regions on the overall experience (Table 1). North American visitors were more likely to rate their experience as excellent (*p* < 0.001). European aquariums, however, had a greater number of terrible ratings than other regions (*p* < 0.05). Higher ratings were given to aquariums with a greater number of comments, with the exception of excellent. The rating of excellent was close to the mean (1489) of comments rather than at the maximum, suggesting that an excellent experience was not necessarily due to the number of comments provided.

### 3.2. Visitor Views on Welfare

#### 3.2.1. Keywords

**Figure 1:**
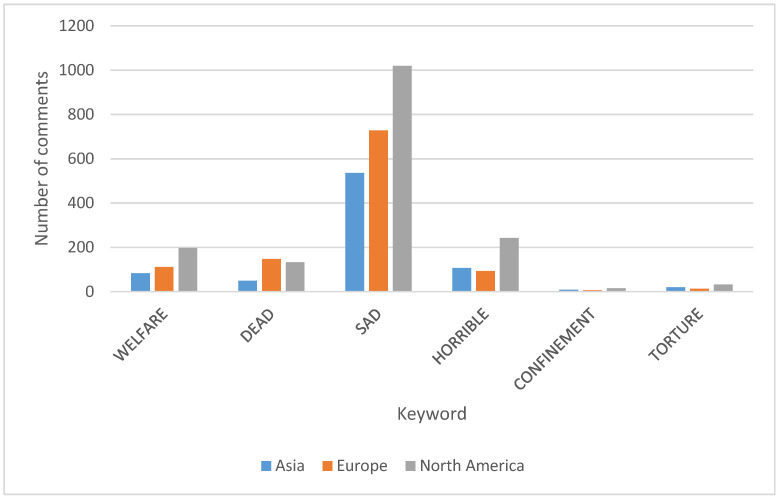
Keywords used within the comments of the three largest regions. The keyword sad had the greatest usage when describing welfare in all three regions. Confinement and torture were not heavily utilized within the comments.

Sad was the most prevalent keyword, appearing 2605 times out of 3984 key words found within the comments. The least-used keyword was confinement (37), followed by torture (72), dead (375), welfare (416) and horrible (479). The word sad was often used as a comment on the status or mood of an animal; for example, penguins were often described as sad, along with dolphins, seals and turtles. The regions that had the greatest number of comments were Asia, Europe and North America. Although having similar number of aquariums within each region, there was a significant difference between the regions with the term sad and other terms that were used (*p* < 0.001) (Figure 1).

#### 3.2.2. Negative Comments

All aquariums had comments from their visitors on TripAdvisor, ranging from their visitor experience, animal experiences and welfare. When just focusing on negative welfare comments, 328 aquariums had at least one comment. Out of the 77 countries, 42% of the countries had at least one negative welfare comment for every aquarium within their country. In contrast, only 10% had no negative welfare comments for the aquariums within their country. However, these were generally represented by countries with just one aquarium present within their borders. The North American region had the highest level of negative comments per number of aquariums (75%) whereas Asia had the fewest (64%). Comments were often related to water quality, tank size or sick fish.

#### 3.2.3. Aquatic Invertebrate Comments

Out of 77 countries, 29 had comments of negative welfare concerning aquatic invertebrates. The number of specific comments, however, were low in number (18%) compared to that of vertebrates (51%) and general welfare (31%) There were no aquatic invertebrate comments within the South American region and limited numbers (*n* = 2) within the African region and Oceania region (*n* = 6). The highest number of comments was in the Europe region (*n* = 43), with the largest number coming from the UK (*n* = 24). North America also showed a high number of comments (*n* = 37), with the largest number coming from a sole country, the USA (*n* = 29).

**Figure 2:**
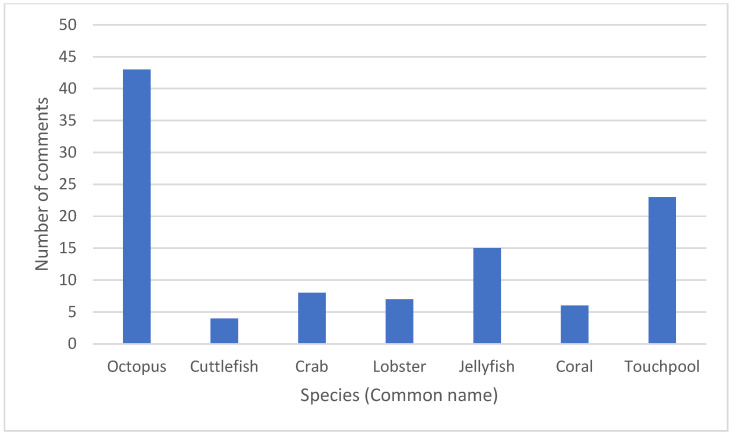
Number of negative welfare comments divided by species; note that touch pools included were containing only invertebrates, with most comments around starfish handling.

When divided into individual species, octopus was mentioned significantly (*p* < 0.01) more than other species (*n* = 43) (Figure 2). Several comments were based around tank size and enrichment of the environment. Invertebrate touch pools were the next highest (*n* = 23), with comments based around excessive force and lifting animals out of the water. Jellyfish had a greater number of comments about being damaged or dead compared to the rest of the invertebrate welfare comments.

The European region had welfare comments in all invertebrate groups. Similarly, the North American region showed similar numbers to Europe except in cuttlefish (*n* = 0) and crab (*n* = 2), where levels of comments were lower. The only regions to have negative comments in respect to crabs were Europe and North America. Asia showed the greatest number of comments in relation to coral welfare (*n* = 4) compared to other regions.

## 4. Discussion

### 4.1. Visitor Perception

By investigating key terms and comments within TripAdvisor, we started to gain insight into the public view of negative aquarium welfare. Emotive words such as sad were used more frequently in comments than the word welfare. The term sad was frequently used in conjunction with particular animals, such as penguins and dolphins. This is possibly due to preconceived ideas around the animals emotional state and vertebrate bias [38]. Species which people connect with on a visit are more likely to incite conservation action for that individual species [41]. It is possible the public may attend to or be specifically interested in certain animals, and therefore are more likely to mention welfare concerns in regard to those animals. Both Europe and North America had a greater number of keywords overall, despite Asia having a similar number of aquariums, suggesting cultural and societal ideology may play a role in how the public perceive welfare. When surveying Chinese and European zookeepers, Bacon et al. [1] found that there was variation in welfare perception; it is possible a similar variation exists within the general public. This may also be reflected in the aquatic invertebrate comments, with corals having a greater representation in Asia than other regions due to their commercial importance for the region.

The overall lower number of poor welfare comments of aquatic invertebrates could be due to numerous factors. Comments concerning vertebrates were emotive, reflecting trends shown by the keywords. It may be that aquatic invertebrates do not elicit the same emotional response in the public. However, if it was lack of emotional involvement, we should see similar numbers within each species, or possibly no comments at all. Instead, we found that octopus received the most comments, with most focused around tank size. This is an interesting observation, as most octopus species have not had their required tank size scientifically investigated. The Giant Pacific Octopus [GPO} Association of Zoos and Aquariums (AZA) tank size is based off aquarists’ experience. Holmberg [42] criticized the lack of scientific rigor when setting the GPO tank size by not utilizing the peer-reviewed literature and only references from aquarists and surveys. By solely using this approach, it does not necessarily account for the animal’s overall need for space, nor does it use the wild populations’ behavior to help inform a tank suitable to exhibit a range of natural behaviors.

Another possibility for the low number of aquatic invertebrate welfare comments may be due to the general understanding of natural behaviors of the animal. This would be particularly evident in species that the public may not have encountered before, and could possibly explain the lower numbers in species such as cuttlefish and coral. Despite showing cognitive ability such as self-control [43] and observational learning, cuttlefish [44] do not receive as much recognition as octopus. If the public were to possibly understand more about cuttlefish cognitive abilities, would they receive similar numbers of comments? It is most likely that a combination of a lack of understanding of welfare cues within the species combined with a familiarity bias has contributed to underrepresentation of comments within this species.

Overall, we could question the reliability of members of the public in identifying welfare concerns, as they are not trained ethologists or even aquarists. Comments including touch pool areas seemed to indicate some understanding of the basic needs of the animals. Comments were around a lack of care from other members of the public or insufficient monitoring by staff. As touch pools are often points of contact with animals, having poor supervision gives a greater potential for animals to be harmed. The harming of animals may be easier for the public to determine even without specialist knowledge of the species. Activities such as lifting animals out of the water or excessive force when touching the animals would be easily identifiable to other members of the public. This may give them more confidence in reporting potential breaches of welfare.

What is potentially interesting is that even if there were comments of poor welfare, it did not necessarily reflect in a lower rating in the aquarium. In some cases, ratings were still 4 out of 5 stars even when commenting on the welfare of an individual organism. People are often drawn to the possibility of having direct and indirect physical contact with a wild animal, which can increase collection appeal [45]. Whether people are willing to compromise on welfare or are aware of the possible implications of direct contact has not been thoroughly investigated. In the North American region, the number of excellent ratings was significantly higher than that of other regions. Despite such high ratings, it was also one of the regions with the highest number of negative welfare comments in relation to aquatic invertebrates and overall.

It is possible that by using TripAdvisor, there may be a country or regional bias introduced. TripAdvisor is an American website and, therefore, the American public may have more familiarity or desire to use it. Analysis of utilization has shown that popularity is actually more global, with European, Oceania, South America and China utilizing the website [33], suggesting that although it may be a factor in commenting, it is less likely to be the sole reason for its use. The UK also showed a greater number of poor welfare comments; however, unlike the USA, they showed the greatest number of terrible ratings of any region. Vasquez [46] suggested that people are more likely to complain online about failure to meet expectations or disappointment than in person, most likely due to the anonymity that they receive. This would possibly explain why welfare comments would be present on a website that is effectively reviewing experiences rather than reporting concerns directly to the aquarium. It is, therefore, suggested that as an aquarium, reviewing negative comments on TripAdvisor would help gain insight into the visitors’ perception of your animal welfare. It would also identify areas where further education is needed for the public to understand welfare indicators; however, to truly achieve this goal, further studies are needed, particularly concerning invertebrates.

### 4.2. Aquarium Welfare Resources

Husbandry guidelines can be invaluable as a starting point to form procedures and welfare indicators. When the number of guidelines is compared between aquatic invertebrates and vertebrates, there is a considerable difference (Table 2). Aquatic invertebrates are underrepresented in all three of the associations examined. In AZA guidelines, aquatic invertebrates and aquatic vertebrates have similar numbers, which are considerably lower than the terrestrial guidelines. The British and Irish Association of Zoos and Aquariums (BIAZA) has a better representation of aquatic invertebrates; however, this may be due to the guidelines being shorter in length and therefore easier to compile. Within the Europe Association of Zoos and Aquariums (EAZA), there are no guidelines available for aquatic invertebrates. Even with terrestrial invertebrates, there are ten times more vertebrate guidelines, even though there are numerous invertebrates within the zoo and aquarium collections. It is, therefore, important that we put greater importance on compiling aquatic invertebrate guidelines. A potential starting point could be to determine welfare indicators while we build husbandry guidelines.

Staff time is often at a premium within aquariums and, therefore, easy-to-implement welfare data collection is vital to success. Understanding of the welfare model and how data collection will be input into welfare decisions may also aid in motivating their collection. Kagan et al. [23] established that conservation and welfare require different priorities, task and goals. It requires almost a mind shift to see how everyday tasks can be incorporated to help advance welfare. By creating a positive environment, the ability to understand the animal better would result in overall standards improving [47]. The human factor of good animal welfare in collections was summarized by Bacon et al. [1], and one of the key findings was that the aquarium should consider the wellbeing of their staff as a function of welfare. Aquarists have a large influence on the organisms that they care for, and if their welfare is compromised then potentially so are the animals. It is important that association bodies also assist in providing guidance and support for data collection. Co-ordination by the associations could also help facilitate collaboration between scientific researchers and aquarists at aquariums.

As our understanding and technology improves, so does the understanding of the animals within our care. Despite this, many aquatic invertebrates remain under investigated, particularly in our understanding of effective states. It is suggested that cognitive bias measurements are adaptable for all species due to the benefit of identifying rewards versus danger [48]. Without the basic understanding of effective states in numerous invertebrate species, being able to use cognitive bias for welfare measurements may be difficult. Using scientific methods such as Go/No-Go tasks and using spatial cues may help start determining bias effects within the lesser-studied aquatic invertebrates [49]. A more scientific approach may also suit the capabilities of those caring for the animals, as it is reported that 88% of USA aquarists have a bachelor’s degree or above [50]. This high level of education would suggest the capacity to learn, collect data, implement it and contribute to invertebrate welfare.

Welfare tool kits could possibly provide standardization and would allow for aquarists to use pre-existing knowledge of the animal. One of the most utilized tool kits within zoos is the Animal Welfare Assessment Grid (AWAG). Its popularity is due to its versatility with different species. The AWAG also allows for a temporal component that many other welfare measures are lacking. A component of research is required for setting up the appropriate indicators and can involve veterinarians, aquarists and the literature. Animal individuality and its possible change over its lifetime gives a versatility that many other models are lacking. In respect to aquatic invertebrates, the AWAG has unfortunately had limited implementation. Narshi et al. [17] successfully demonstrated the use of the AWAG on cephalopods and decapods, although they suggested further study and utilization is needed to test its full potential for aquatic invertebrates. By investigating aquatic invertebrate species utilizing the AWAG, data can be generated and tested over multiple collections. In addition, the sharing of data and indicators is something that zoo and aquarium associations could co-ordinate. It is this collaborative aspect within the aquarium community that will help increase our understanding of aquatic invertebrate welfare.

## 5. Conclusions

A knowledge gap still exists around aquatic invertebrate welfare in public aquaria. Awareness of negative welfare indicators by the public is limited to a few species and often emotive when being discussed. TripAdvisor may offer an insight into the public’s overall understanding and opinions of welfare within an aquarium due to the anonymity. Europe and North American regions have a greater number of comments in respect to aquatic invertebrate welfare compared to other regions.

Aquarium and zoo associations should be encouraged to help facilitate and produce guidelines for more aquatic invertebrate species. By making use of the scientific and veterinary communities, welfare indicators in welfare tool kits can become more robust. In addition, incorporating input from invertebrate aquarists are vital for improving overall standards if we are to have greater representation of invertebrate welfare in public aquaria. As such, aquarium collections need to support and train their staff in welfare tools and consider their role in championing welfare.

## Figures and Tables

**Table 1:** Overall customer satisfaction ratings divided by geographic regions. Values are given in percentages for the overall ratings (Terrible, Poor, Average, Very good, Excellent).

Region	Terrible	Poor	Average	Very Good	Excellent
Africa	4.7	4.1	19.8	36.5	34.9
Asia	3.7	5.3	19.0	35.0	37.0
Europe	6.3	9.4	20.2	28.8	35.3
North America	2.1	3.3	12.5	30.7	51.4
Oceania	2.1	5.4	18.1	36.3	38.1
South America	4.5	3.8	18.9	34.6	38.1

**Table 2:** Zoo associations and the number of produced guidelines as of February 2023.

Association	Terrestrial Vertebrates	Terrestrial Invertebrates	Aquatic Vertebrates	Aquatic Invertebrates
AZA	28	0	3	3
BIAZA	44	17	12	7
EAZA	50	5	5	0
Total	132	22	20	10

## Data Availability

The data presented in this study are available on request from the corresponding author.
